# Prognostic value of HbA1c for in-hospital and short-term mortality in patients with acute coronary syndrome: a systematic review and meta-analysis

**DOI:** 10.1186/s12933-019-0970-6

**Published:** 2019-12-11

**Authors:** Wenjun Pan, Haining Lu, Baotao Lian, Pengda Liao, Liheng Guo, Minzhou Zhang

**Affiliations:** 1 Guangzhou University of Chinese Medicine, Guangzhou, 510405 China; 2 The Second Clinical College of Guangzhou University of Chinese Medicine, Guangzhou, 510120 China; 3 Guangdong Provincial Hospital of Chinese Medicine, No 111 Dade Road, Guangzhou, 510120 China

**Keywords:** Glycated hemoglobin A, Acute coronary syndrome, Mortality, Predictor

## Abstract

**Background:**

HbA1c, the most commonly used indicator of chronic glucose metabolism, is closely associated with cardiovascular disease. However, the relationship between HbA1c and the mortality of acute coronary syndrome (ACS) patients has not been elucidated yet. Here, we aim to conduct a systematic review assessing the effect of HbA1c on in-hospital and short-term mortality in ACS patients.

**Methods:**

Relevant studies reported before July 2019 were retrieved from databases including PubMed, Embase, and Central. Pooled relative risks (RRs) and the corresponding 95% confidence interval (CI) were calculated to evaluate the predictive value of HbA1c for the in-hospital mortality and short-term mortality.

**Results:**

Data from 25 studies involving 304,253 ACS patients was included in systematic review. The pooled RR of in-hospital mortality was 1.246 (95% CI 1.113–1.396, p: 0.000, I^2^ = 48.6%, n = 14) after sensitivity analysis in studies reporting HbA1c as categorial valuable. The pooled RR was 1.042 (95% CI 0.904–1.202, p: 0.57, I^2^ = 82.7%, n = 4) in random-effects model for studies reporting it as continuous valuable. Subgroup analysis by diabetic status showed that elevated HbA1c is associated increased short-term mortality in ACS patients without diabetes mellitus (DM) history and without DM (RR: 2.31, 95% CI (1.81–2.94), p = 0.000, I^2^ = 0.0%, n = 5; RR: 2.56, 95% CI 1.38–4.74, p = 0.003, I^2^ = 0.0%, n = 2, respectively), which was not the case for patients with DM and patients from studies incorporating DM and non-DM individuals (RR: 1.16, 95% CI 0.79–1.69, p = 0.451, I^2^ = 31.9%, n = 3; RR: 1.10, 95% CI 0.51–2.38), p = 0.809, I^2^ = 47.4%, n = 4, respectively).

**Conclusions:**

Higher HbA1c is a potential indicator for in-hospital death in ACS patients as well as a predictor for short-term mortality in ACS patients without known DM and without DM.

## Introduction

Acute coronary syndrome (ACS) is the leading cause of death worldwide. Although the prognosis of ACS patients has been improved in the past 18 years, there is a lot of room for further improvement, as the ACS patients clearly have a higher risk for a recurrent cardiovascular events compared with the corresponding general population [1]. Biomarkers are good for prognosis evaluation and risk stratification of ACS patients. Cardiac troponins, CRP and NT- BNP are the main predictors for ACS patients. However, they still have some drawbacks as BNP levels are also found to be significantly influenced by age, fluid loading and physical exercise [2], and CRP is influenced by inflammation. Cardiac troponins are affected by reperfusion modalities [3]. There is a need for more reliable biomarkers for the prognosis of ACS patients.

Hyperglycemia and newly-diagnosed diabetes mellitus (DM) are found in a large number of ACS patients and a strong predictor for the poor prognosis of these patients [4–9]. HbA1c, which reflects average blood glucose concentrations over the previous 8–12 weeks, was shown to be a better predictor of prognosis following ACS than fasting and admission glucose [10]. Recent studies proved that HbA1C is an independent predictor of mortality [11], while other studies got opposite results [12]. Furthermore, intensive glycemic control using insulin injection did not improve both short-term and long-term mortality in patients with ACS and DM [13, 14]. In spite of current uncertainty, no previous systematic review has summarized the prognostic role of HbA1c playing in ACS patients. We therefore conducted the meta-analysis of prospective and retrospective cohort studies to evaluate if high levels of HbA1c are associated with increased in-hospital and short-term mortality in ACS patients.

## Methods

### Search strategy

To find potentially relevant studies, we searched articles in English referenced in Medline, Central and Embase from database inception until July, 2019. The following free texts or MeSH terms were used in search: Acute coronary syndrome, unstable angina, ST-elevation, ST-segment, STEMI, NSTEMI or myocardial infarction combined with hemoglobin a1c, HbA1c, glycated hemoglobin, glycosylated hemoglobin, haemoglobin a1c, glycated haemoglobin, glycosylated haemoglobin, glycohemoglobin a or glycohaemoglobin a. The reference lists of retrieved articles were reviewed for additional eligible studies. Eligibility and study selection

Two authors (Wenjun, Haining) independently reviewed the titles and abstracts of all articles retrieved, and selected those meeting the following criteria: (1) Based on prospective and retrospective cohort studies. (2) Conducted in adults aged 18 years or older. (3) Reporting HbA1c levels as exposure. (4) Reporting the in-hospital and short-term (less than 1 year) mortality. (5) Reporting the effect of the exposure in the form of a relative risk [risk ratio (RR), odds ratio (OR) or hazard ratio (HR)] and their 95% CI or the dead number and total number of each group. Cross-sectional and case–control studies were excluded.

### Data extraction and quality assessment

Two investigators (Wenjun and Baotao) independently extracted the following information from eligible studies: first authors’ name, publication year, study name, country, age range and/or mean age (years), number of participants, exposure levels, RRs and their 95% CI as well as the alive and dead numbers for each exposure category. We selected the RRs from the model with the most comprehensive covariate adjustment, and used the Newcastle–Ottawa Scale to assess the quality of the studies included [15]. Any discrepancies were resolved through discussion under supervision of a senior author (Minzhou).

### Statistical analysis

We calculated the average RRs for the top compared with the bottom quantile of HbA1c levels using a fixed-effects model. We calculated the unadjusted RRs using original data published in the studies if RRs and their 95% CI were not available in the papers. The I^2^ statistic and Cochran’s Q test were used to evaluate heterogeneity. If there is significant heterogeneity (I^2^ > 50%, p < 0.1) among studies, we conducted sensitivity analysis or subgroup analysis to explore the sources of heterogeneity. Publication bias were assessed using funnel plots’ asymmetry and tested with Egger’s asymmetry test and Begg’s test (p < 0.05) when there were at least 10 studies. All analysis was conducted with Stata 13.0.

## Results

### Search results

**Fig. 1:**
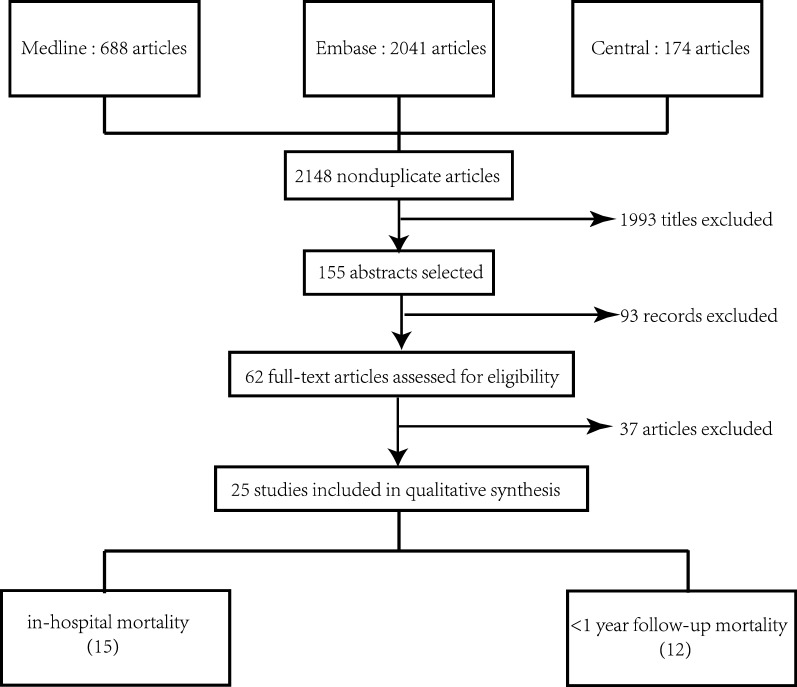
Flowchart of study search and identification

The search strategy is presented in Fig. 1. The literature search initially identified 2148 publications. After screening the titles, the number was reduced to 155 after eliminating randomized controlled trials regarding therapy, reviews and animal experiments, or irrelevant to the current analysis. After screening the remaining abstracts, there were 62 papers for full-text reading, which yielded 25 studies involving 26 papers for final analysis. There are 18 studies exploring the role of HbA1c in in-hospital mortality, and 13 studies regarding less than one-year follow-up mortality.

### Study characteristics

Table 1 shows the characteristics of these 25 studies from 1987 to 2019 involving 304,253 ACS patients. The mean age of patients in the included studies ranged from 50.9 to 70.1 years old. The male percentage of each studies ranged from 53.6 to 81.7%. Participants had diverse metabolic status including DM, without known DM, without DM and no specified glucose metabolic status. Most of included studies were with high quality with the average quality score as 8.27.

**Table 1:** Characteristics of included studies

First author	Year	Number screened	Age at screening	Men (%)	Follow-up	Glucose metabolic state	Country/continent	Quality score
Oswald/Yudkin [16, 33]	1987/1988	397	64.6	76	3 months/in hospital	AMI without known DM	UK/Europe	8
Cao [17]	2005	349	NA	NA	In hospital	AMI with DM	US/America	6
Rasoul [34]	2007	504	63	71.8	1 month	STEMI without known DM	Netherlands/Europe	9
Cakmak [35]	2008	100	59.6	68	28 days	AMI	Turkey/Europe	8
Chan [19]	2010	317	70	53.6	6 months/in hospital	ACS with DM	China/Asia	8
Timmer [36]	2011	4176	62.2	74	30 days	STEMI without known DM	Netherlands/Europe	9
Britton [18]	2011	16,004	64.7	61.2	In hospital	AMI with known DM	US/America	9
Cicek [20]	2011	374	55.9	85	In hospital	STEMI	US/America	9
Lazzeri [21]	2011	195	70.1	67.2	In hospital	STEMI and DM	Italy/Europe	9
Lazzeri [22]	2012	518	NA	NA	In hospital	STEMI without DM	Italy/Europe	9
Liu [23]	2012	4793	62.6	71.5	30 days/7 days	STEMI	China/Asia	9
Ahmad [37]	2012	754	52.0	67.9	1 month	STEMI	Pakistan/Asia	7
Tian [38]	2013	608	61.4	79.1	30 days	AMI	China/Asia	9
Pusuroglu [25]	2014	443	54.7	81.7	1 month/in hospital	STEMI	Turkey/Europe	7
Blasco [24]	2014	601	62	78	In hospital	AMI without known DM	Spain/Europe	9
Fujino [31]	2014	696	67.7	72	In hospital	AMI	Japan/Asia	7
Rousan [26]	2014	243,861	64	65.1	In hospital	AMI	US/America	9
El-sherbiny [39]	2015	60	57.8	80	6 months	STEMI without known DM	Egypt/Africa	8
AbuShady [27]	2015	151	50.9	70.5	In hospital	ACS without known DM	Egypt/Africa	8
Aggarwal [28]	2016	1686	60.4	67.4	In hospital	STEMI	US/America	9
Donghun[30]	2016	2470	61.9	77.5	In hospital	STEMI without DM	Korea/Asia	9
Samir [29]	2016	208	55.9	54.8	6 months/in hospital	STEMI without DM	Egypt/Africa	9
Heller [40]	2017	5380	61	67.9	1 month	ACS with T2DM	UK/Europe	9
Kim [32]	2017	12,625	64	73.9	In hospital	AMI	Korea/Asia	7
Hermanides [40]	2019	6586	65.0	71.2	30 days	AMI without known DM	Netherlands/Europe	7

*AMI* acute myocardial infarction, *DM* diabetes mellitus, *STEMI ST* segment elevated myocardial infarction, *ACS* acute coronary syndrome, *NA* not available

The baseline characteristics and therapy after admission of the highest and lowest HbA1c groups were summarized in Tables 2 and 3. There were more patients with hypertension in the highest HbA1c group, which might account for the increased number of patients taking ACEI (angiotensin-converting enzyme inhibitors) or ARB (angiotensin receptor blocker) before and after admission. However, patients with the lowest HbA1c levels had a higher hyperlipidemia incidence, resulting in the increased number of patients taking lipid lowering drugs at baseline. The adherence to antiplatelet in either group showed no difference, but patients in the highest HbA1c group got more PCI (percutaneous coronary intervention) and CABG (coronary artery bypass grafting) therapies according to their medication history. Based on the Killip grade at admission, more ACS patients had cardiac dysfunction (Killip ≥ 2) in the highest HbA1c group compared to the lowest ones, but the number of patients with severe cardiac dysfunction (Killip ≥ 3) showed no difference, indicating patients in the highest HbA1c group had worse cardiac function at admission. Patients had a higher revascularization rate in the lowest HbA1c group. Data from 4 trials showed higher percentage of ACS patients taking antiplatelet agents in highest HbA1c group while in the other three studies it showed the opposite results.

**Table 2:** Baseline characteristics of the lowest and highest HbA1c group

Baseline characterization	Lowest	Highest	Total number	
			Lowest	Highest
Male (%), studies = 19	71.69%	73.45%	45,354	10,909
Age (year), studies = 19	61.25602	62.03319	45,354	10,909
Smoker (%), studies = 15	42.25%	39.54%	43,277	9681
BMI (kg/m^2^), studies = 11	25.87935	26.00163	283	301
BMI > 30 kg/m^2^ (%), studies = 2	21.15%	22.22%	52	63
Previous MI (%), studies = 11	16.73%	15.57%	39,307	8737
Hypertension (%), studies = 15	38.52%	43.27%	9157	4708
Heart failure (%), studies = 3	5.24%	5.79%	36,565	6925
Cerebrovascular accident (%), studies = 6	4.96%	5.58%	37,262	7281
Hyperlipidemia (%), studies = 10	47.72%	41.92%	41,808	7855
Renal insufficiency (%), studies = 3	26.47%	25.35%	774	447
Antiplatelet (%), studies = 2	17.47%	20.54%	596	294
Aspirin (%), studies = 2	34.50%	33.60%	35,325	5952
Clopidogrel (%), studies = 2	7.69%	8.35%	35,325	5952
PCI (%), studies = 6	8.42%	10.78%	3031	2283
CABG (%), studies = 4	2.43%	4.08%	1745	1323
ACEI/ARB (%), studies = 4	27.97%	34.02%	36,013	6276
Lipid lowering drugs (%), studies = 4	27.76%	25.24%	36,013	6276
Beta-blocker (%), studies = 3	26.39%	27.11%	35,901	6194
Creatinine (mg/dl), studies = 4	1.259,418	1.105723	1388	1839
eGFR (mg/dl/1.73 m^2^), studies = 5	87.00478	68.16183	5507	1654
Admission plasma glucose, studies = 11	7.84536	9.461979	4831	4171
Killip ≥ 3 (%), studies = 3	11.75%	14.33%	1081	1584
Killip ≥ 2 (%), studies = 8	9.88%	16.68%	5782	1915

*BMI* body mass index, *MI* myocardial infarction, *PCI* percutaneous coronary intervention, *CABG* coronary artery bypass grafting, *ACEI* angiotensin-converting enzyme inhibitors, *ARB* angiotensin receptor blocker, *eGFR* estimated glomerular filtration rate

**Table 3:** Therapy after admission of the lowest and highest HbA1c groups

Therapy after admission	Lowest (%)	Highest (%)	Total number	
			Lowest	Highest
Thrombosis (%), studies = 3	7.40	11.86	36,328	6808
PCI (%), studies = 13	94.86	88.92	42,985	9124
PCI (successful) (%), studies = 6	92.99	90.05	5477	3114
CABG (%), studies = 3	19.90	16.44	39,291	6326
Antiplatelet agent (%), studies = 4	78.44	84.25	4058	584
Aspirin (%), studies = 3	98.46	97.88	36,565	6925
Clopidogrel or Tigrilo (%), studies = 3	72.32	69.16	36,565	6925
Lipid lowering drugs (%), studies = 7	92.74	89.93	40,623	7509
Beta blockers (%), studies = 7	94.79	90.98	40,623	7509
ACEI/ARB (%), studies = 7	73.92	77.34	40,623	7509

*PCI* percutaneous coronary intervention, *CABG* coronary artery bypass grafting, *ACEI* angiotensin-converting enzyme inhibitors, *ARB* angiotensin receptor blocker

### In-hospital mortality

**Fig. 2:**
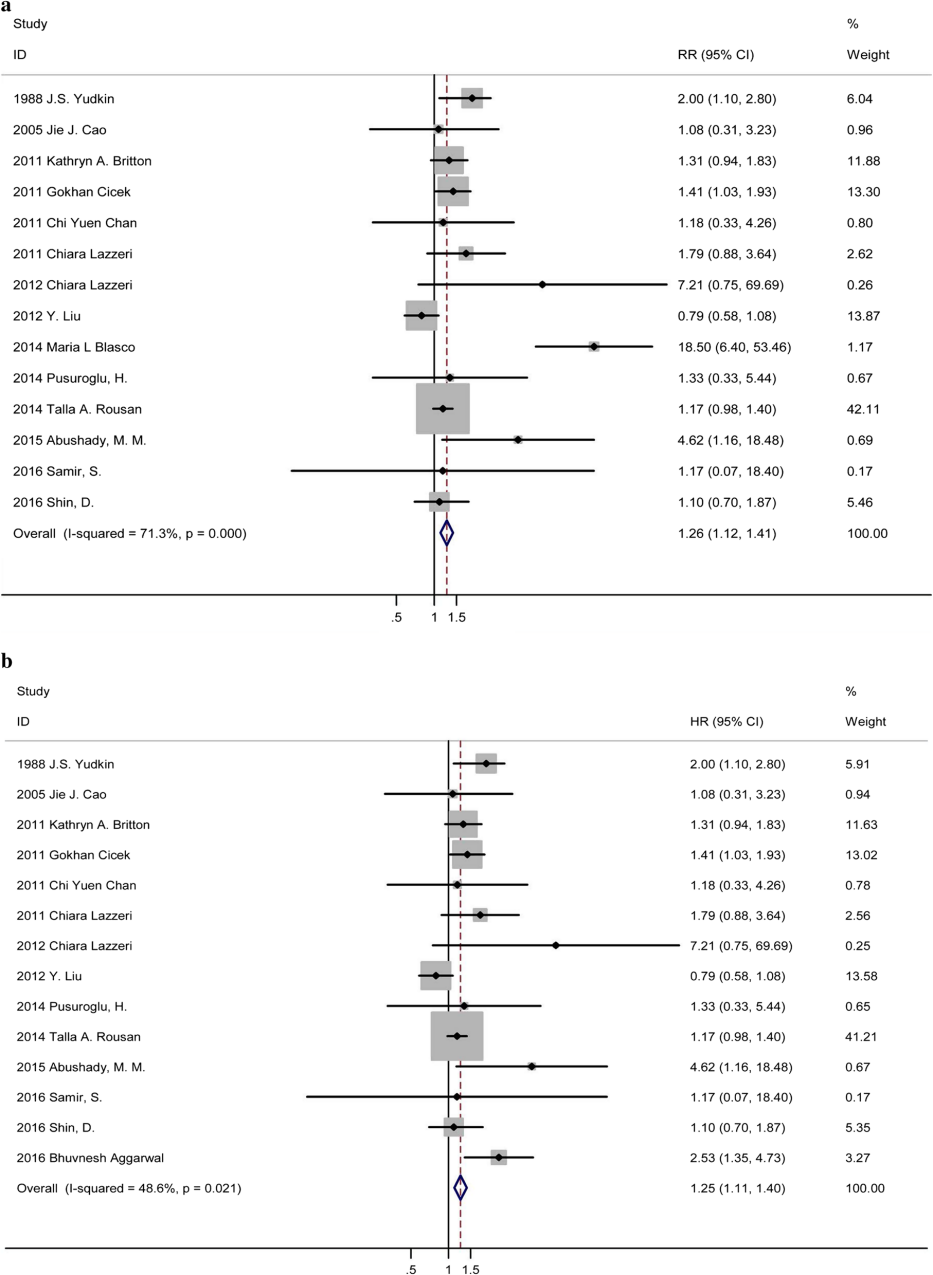
**a** Forest plot of categorial valuable HbA1c and relative risk of in-hospital mortality among ACS patients. **b** Forest plot of categorial valuable HbA1c and relative risk of in-hospital mortality among ACS patients after sensitivity analysis

Fifteen studies referred HbA1c as a categorial valuable [16–30], and showed that when compared to lower HbA1c groups, higher HbA1c was significantly associated with increased in-hospital mortality (RR: 1.285, 95% CI 1.148–1.439, p: 0.000, I^2^ = 71.9%, n = 15) (Fig. 2a). Due to the significant heterogeneity, a sensitive analysis was applied. Result from the other 14 studies indicated that a significant positive relationship persisted after excluding the study causing the remarkable heterogeneity [24] (RR: 1.246, 95% CI 1.113–1.396, p: 0.000, I^2^ = 48.6%, n = 14) (Fig. 2b). No publication bias was found using Begg’s and Egger’s test (p > 0.05). Four studies referred HbA1c as continuous variable [18, 23, 31, 32], and they all reported RR in multivariable analysis. The pooled RR was 1.058 (95% CI 1.012–1.105, p: 0.012, I^2^ = 82.7%, n = 4) in fixed-effects model. The sensitivity analysis suggested that the heterogeneity did not originate single studies. Using random-effects model, there is no significant relationship between HbA1c levels and in-hospital mortality (RR: 1.042, 95% CI 0.904–1.202, p: 0.57, I^2^ = 82.7%, n = 4).

### Less than 1-year follow-up mortality

**Fig. 3:**
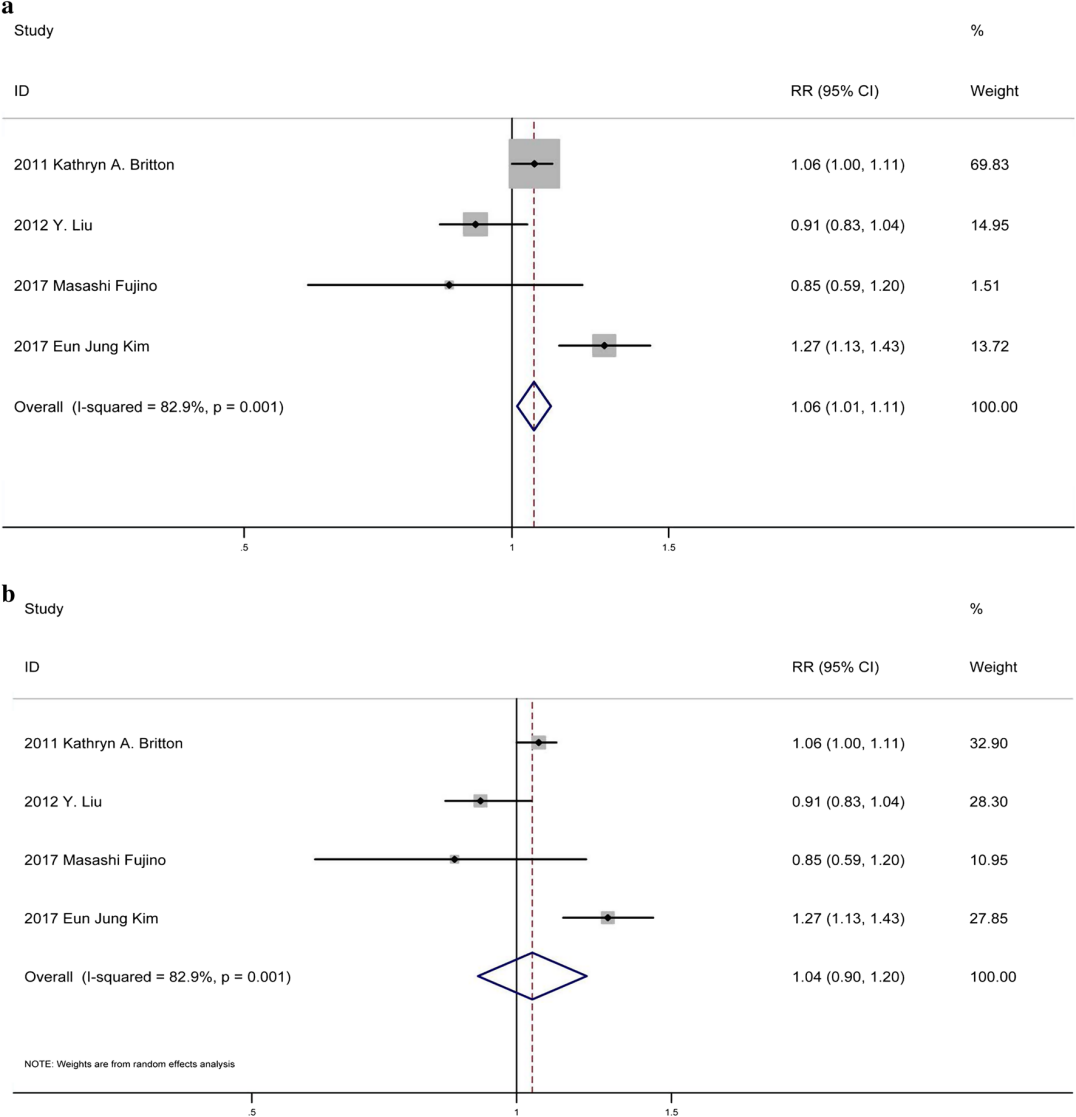
**a** Forest plot of continuous valuable HbA1c and relative risk of in-hospital mortality among ACS patients using fixed-effects model. **b** Forest plot of continuous valuable HbA1c and relative risk of in-hospital mortality among ACS patients in random-effects model

**Fig. 4:**
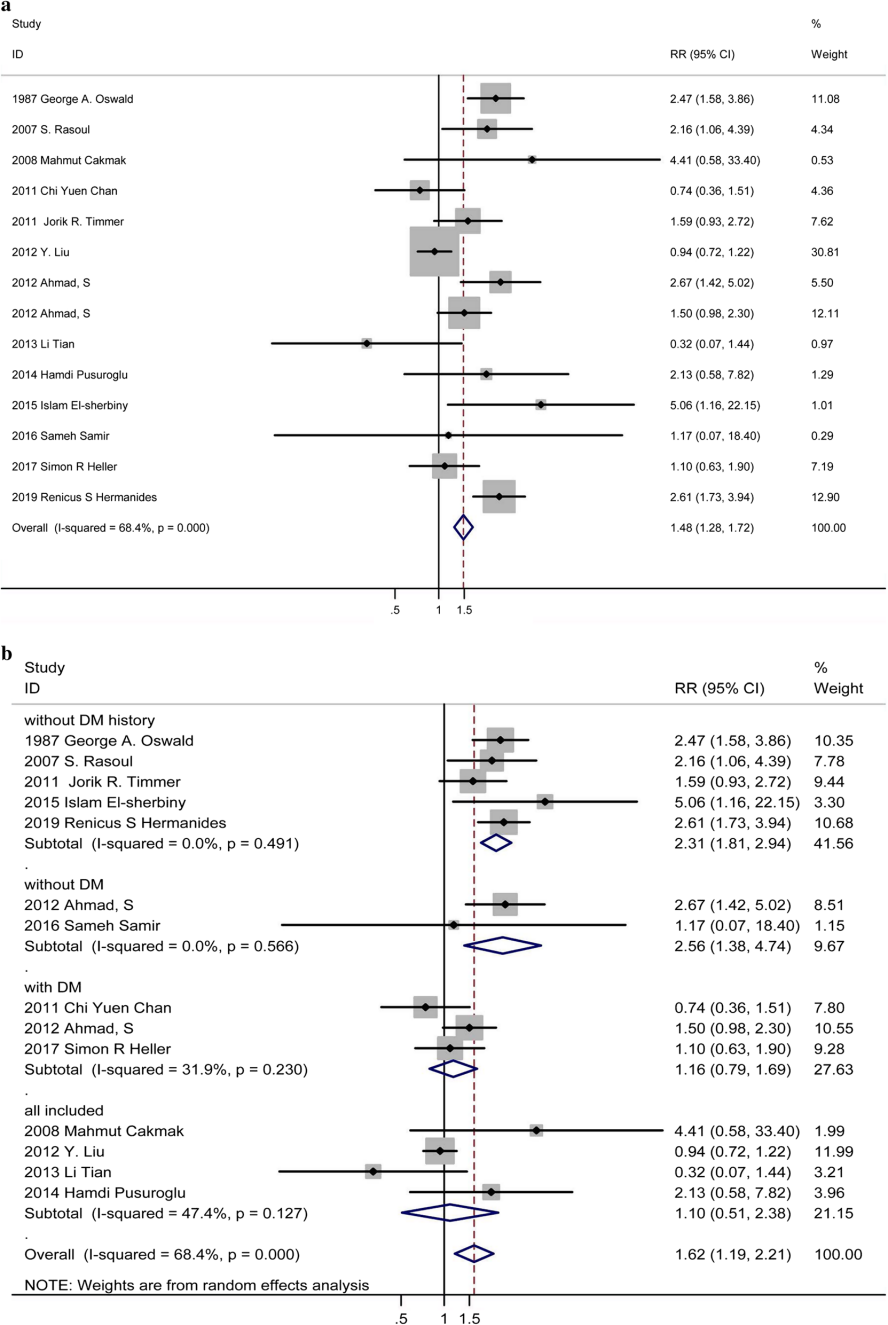
**a** Forest plot of HbA1c and relative risk of short-term mortality among ACS patients. **b** Forest plot of HbA1c and relative risk of short-term mortality among ACS patients by subgroup analysis

Thirteen studies [19, 23, 25, 29, 33–41] showed that incremental HbA1c levels were associated with an obviously increased risk of mortality (RR: 1.48, 95% CI 1.28–1.72, p: 0.002, I^2^ = 68.4%, n = 13) (Fig. 3a). Because heterogeneity test showed that there were significant differences between individual studies, we conducted subgroup analysis according to different diabetic status. Subgroup analysis, dividing the trials into four subgroups including ACS patients without DM history, without DM, with DM, and incorporated with and without DM (Fig. 4b). Patients without DM history in subgroup one is patients without previous diagnosis of DM or treatment with a diet, oral glucose-lowering medication and/or insulin. Patients with DM included patients with previous diagnosis of DM or on the anti-diabetic treatment as well as newly diagnosis of DM at admission based on the criteria of WHO (World Health Organization) or ADA (American Diabetes Association). Patients without DM excluded patients with previous or newly diagnosis of DM based on the criteria above. All included means original studies incorporated patients with and without DM. As shown in Fig. 4b, the RR of all-cause mortality in patients without DM history at admission in subgroup one was 2.31 [95% CI (1.81–2.94), p = 0.000], with little heterogeneity as I^2^ = 0.0%. The RR of all-cause mortality in patients without DM at admission in the subgroup two was 2.56 [95% CI 1.38–4.74, p = 0.003], with little heterogeneity I^2^ = 0.0%. For patients with DM at admission, the RR of all-cause mortality was 1.16 [95%CI (0.79,1.69), p = 0.451], with small heterogeneity I^2^ = 31.9%. For ACS patients with incorporated diabetic status, the RR of all-cause mortality was 1.10 [95% CI (0.51, 2.38), p = 0.809], with acceptable heterogeneity I^2^ = 47.4%. The analysis suggested that higher HbA1c in ACS patients without DM history or without DM at admission was significantly related to increased short-term all-cause mortality, but not for ACS patients with DM or not specific metabolic status. No publication bias was found among the studies (p > 0.05) (Additional file 1: Figure S1b).

## Discussion

25 clinical trials were included in this study involving 304,253 subjects. We found: (1) higher HbA1c levels contributed to increased in-hospital mortality in ACS patients; (2) in general, HbA1c was significantly associated with increased short-term mortality in ACS patients; (3) for ACS patients without DM history and without DM, higher HbA1c had an positive relationship with increased short-term mortality; (4) for ACS patients with DM and no specified metabolic status, HbA1c was failed to show positive prediction for short-term mortality.

Consistent to the review by Capes et al. [42], we found positive relationship between admission hyperglycemia and in-hospital mortality in ACS patients for studies considering HbA1c as a categorial valuable. Although there was significant heterogeneity among studies which came from the one with the highest RR value, the association between HbA1c levels and in-hospital mortality remained significant after excluding it. As compared to other studies, the in-hospital mortality (14%) was higher in the top quantile of HbA1c, which might cause the great heterogeneity. For studies considering HbA1c as a continuous valuable, in fixed-effects model we found a positive relationship between HbA1c and in-hospital mortality but failed to prove it in the random-effects model. Besides the limited number of studies, the diverse valuables for adjusted in the multivariable analysis should also be taken into consideration. As reported by several independent groups, hyperglycemia is associated with impaired microvascular function and decrease coronary flow velocity even induce microvascular obstruction in ACS patients [43–45]. No reflow after reperfusion therapy due to chronic hyperglycemia could give rise to in-hospital death in ACS patients. We did pooled analysis of all the studies of short-term mortality and found that HbA1c contributes to increased short-term mortality in ACS patients. However, there was significant heterogeneity among these studies, which was resolved through subgroup analysis according to metabolic status. We found that for ACS patients without known DM, HbA1c significantly increases short-term mortality. For these included studies, besides patients with normal glucose metabolic status, there was high incidence of patients with newly diagnosed DM which have not been recognized and treated [46]. Therefore, this subgroup reflects the effect of HbA1c on short-term mortality independent of previous anti-diabetic drugs. However, consistent with Østergaard et al. [47] and She et al. [48], we found for patients with DM, it showed no significant effect of HbA1c on short-term mortality in ACS patients. Several causes should be taken into consideration. Firstly, there might be several confounding factors interfering the results, such as anti-diabetic drugs, modified lifestyle and better compliance to the doctors’ advice before and after heart attack. Secondly, as reported by Li et al. [49], higher HbA1c were associated with less risk of myocardial injury following PCI in diabetic patients because of better energy supply. As a large number of ACS patients included in our meta-analysis received PCI therapy, this might account for the confounding factor in short-term prognosis. Last but not least, a limited number of patients with a relatively short follow-up should be taken into consideration, as HbA1c has been commonly recognized as a long-term predictor of mortality in ACS patients with DM [50, 51]. Consistent with randomized controlled trials [13, 14], these patients did not benefit from intensive glycemia control after ACS. The intriguing thing we found in this systematic review is that for ACS patients with definitely no DM, HbA1c is also showed to be a predictor for short-term mortality, which is consistent with the finding by Geng et al. [52]. Overall, HbA1c is a predictor for in-hospital and short-term death in ACS patients. For DM and not specified patients, due to complicate confounding factors which might interfere the results, we found no positive relationship between HbA1c levels and short-term mortality. More studies regarding HbA1c as a continuous variable are needed to prove its effect on in-hospital mortality.

A number of biological mechanisms have been proposed to explain a potential causal association between HbA1c and increased mortality in ACS patients. Firstly, basic experimental study indicates that hyperglycemia inhibits cardiac stem cells from cardiac repair and angiogenesis in streptozotocin-induced diabetic mice undergoing AMI surgery [53], which might account for the worse prognosis for ACS patients with elevated HbA1c levels. Secondly, higher HbA1c levels reflect over-glycosylation of intracellular protein in cardiac myocytes like CaMKII [54]. This may give rise to sudden cardiac death due to fatal arrhythmia. Furthermore, as reviewed by Shah et al. [55], chronic hyperglycemia causes decreased mitochondrial biogenesis, increased reactive oxygen species (ROS) production and post-translational protein modifications, which eventually leads to malignant cardiac remodeling. Last but not least, recent experiment found that baseline plasma fibrinogen is associated HbA1c levels in ACS patients [56]. Fibrinogen is an important part of coronary thrombosis, which is consistent with that higher HbA1c levels were significantly related to increased refraction in ACS patients [25, 27, 41].

The present meta-analysis has some inevitable limitations. On one hand, for the categorial variables, we only took the highest and the lowest quantiles into consideration, which might exaggerate the effect of HbA1c. On the other hand, while we extracted the adjusted RRs if they are available, as we cannot get individual participant data, the confounding factors such as baseline characterization and therapy after admission might still interfere the results. Thus, the result should be interpreted with caution in clinical practice.

While acknowledging the inherent limitations of original data, we insist there are merits and clinical significance of our meta-analysis. To our knowledge, this is the first systematic review regarding the predictive value of HbA1c for short-term mortality in ACS patients. Although there is a similar review regarding STEMI patients [57], it did not explain the heterogeneity explicitly. As unstable angina and NSTEMI share the same pathological process as STEMI, we took all the ACS patients into account in this systematic review. Due to the increased in-hospital mortality in patients with elevated HbA1c, people at high risk of ACS should take plasma glucose and HbA1C under control. According to recent reports, there are substantial ACS patients with unknown DM at admission [34, 41], which accounts for the high risk of short-term mortality in ACS patients without known DM. It is intriguing to note that elevated HbA1c is a predictor of short-term mortality in ACS patients without DM, which instructs ACS patients especially pre-DM patients to control the plasma glucose and HbA1c levels strictly [13, 14]. While there are randomized controlled trials indicating that ACS patients with DM did not benefit from the intensive glycemia control, we recommend people at high risk of ACS without (known) DM to monitor and control HbA1c levels who might benefit from it according to our meta-analysis.

## Conclusion

Elevated HbA1c is a predictor of in-hospital mortality in ACS patients and short-term mortality in ACS patients without known DM and without DM. HbA1c is a stable indicator of long-term glucose control and insulin resistance, which should be prevalently monitored by people at high risk of ACS.

## Supplementary information

**Additional file 1: Figure S1.** a Funnel plot of categorial valuable HbA1c and relative risk of in-hospital mortality among ACS patients. b Funnel plot of HbA1c and relative risk of short-term mortality among ACS patients

## Data Availability

All data generated or analyzed during this study are included in this published article [and its additional information files]. Not applicable.

## Abbreviations

ACS: acute coronary syndrome
RR: relative risk
OR: odds ratio
HR: hazard ratio
CI: confidence interval
DM: diabetes mellitus
CRP: C reactive protein
BNP: B-type natriuretic peptide
ROS: reactive oxygen species
AMI: acute myocardial infarction
STEMI: ST-segment elevated myocardial infarction
NSTEMI: non ST-segment elevated myocardial infarction
ACEI: angiotensin-converting enzyme inhibitors
ARB: angiotensin receptor blocker
BMI: body mass index
PCI: percutaneous coronary intervention
CABG: coronary artery bypass grafting
eGFR: estimated glomerular filtration rate
WHO: World Health Organization
ADA: American Diabetes Association
