# Supplementary figures and images for: Prognostic value of HbA1c for in-hospital and short-term mortality in patients with acute coronary syndrome: a systematic review and meta-analysis
Source: Cardiovasc Diabetol. 2019 Dec 11;18:169. doi: 10.1186/s12933-019-0970-6 (PMC6905004; doi:10.1186/s12933-019-0970-6)

a

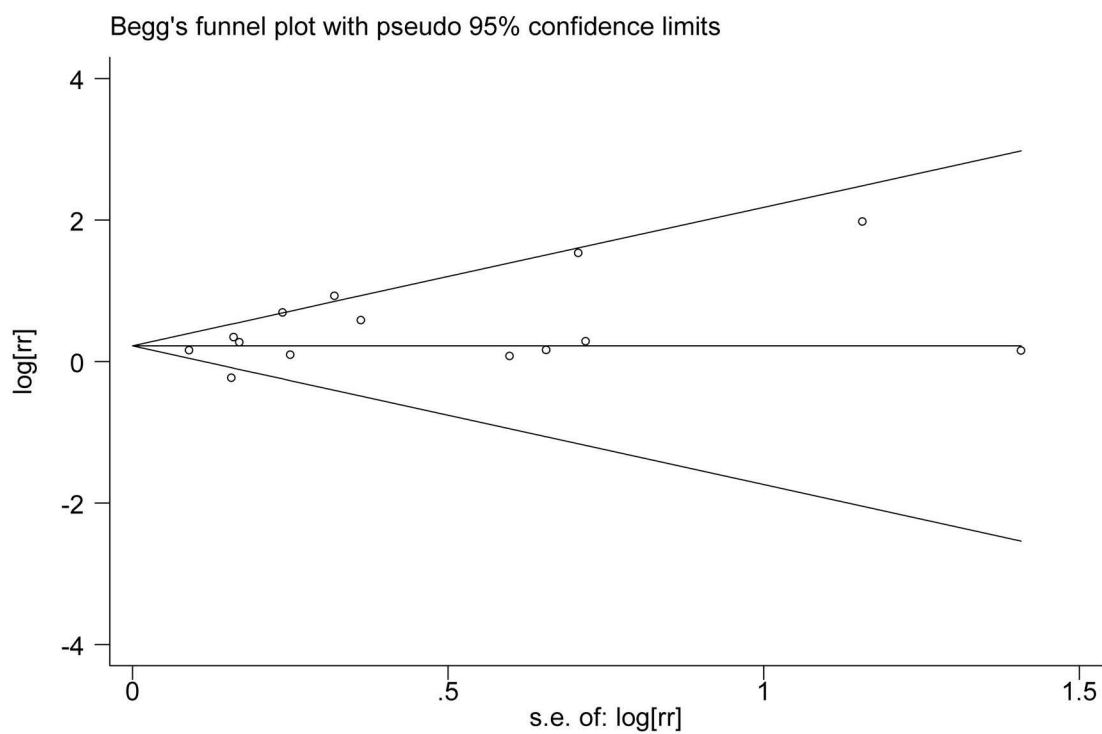

b

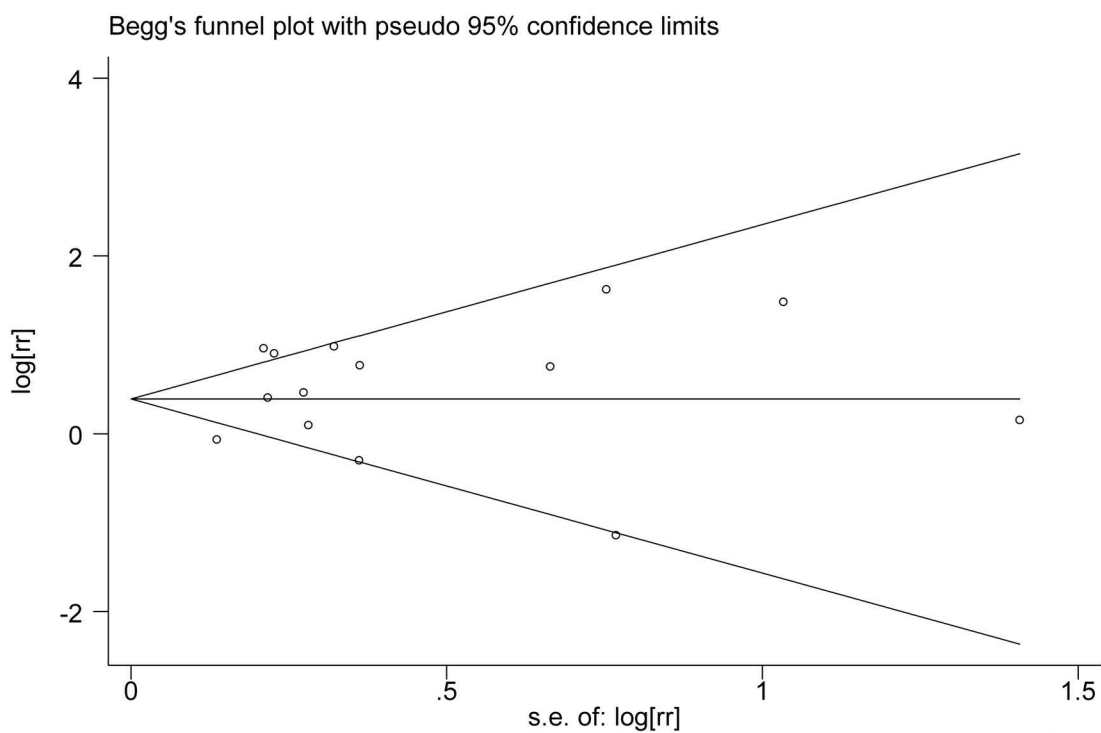

Supplement: Supplementary file 1 — Additional file 1: Figure S1. a Funnel plot of categorial valuable HbA1c and relative risk of in-hospital mortality among ACS patients. b Funnel plot of HbA1c and relative risk of short-term mortality among ACS patients [file 12933_2019_970_MOESM1_ESM.pdf]
